# Mapping Monthly Water Scarcity in Global Transboundary Basins at Country-Basin Mesh Based Spatial Resolution

**DOI:** 10.1038/s41598-018-20032-w

**Published:** 2018-02-01

**Authors:** Dagmawi Mulugeta Degefu, He Weijun, Liao Zaiyi, Yuan Liang, Huang Zhengwei, An Min

**Affiliations:** 1 College of Hydraulic and Environmental Engineering, China Three Gorges University, P.O. Box 443002 Yichang, China; 2 College of Economics and Management, China Three Gorges University, P.O. Box 443002 Yichang, China; 3 Faculty of Engineering and Architectural Science, Ryerson University, P.O. Box M5B 2K3 Toronto, Canada; 4 School of Business, Hohai University, P.O. Box 211000 Nanjing, China

## Abstract

Currently fresh water scarcity is an issue with huge socio-economic and environmental impacts. Transboundary river and lake basins are among the sources of fresh water facing this challenge. Previous studies measured blue water scarcity at different spatial and temporal resolutions. But there is no global water availability and footprint assessment done at country-basin mesh based spatial and monthly temporal resolutions. In this study we assessed water scarcity at these spatial and temporal resolutions. Our results showed that around 1.6 billion people living within the 328 country-basin units out of the 560 we assessed in this study endures severe water scarcity at least for a month within the year. In addition, 175 country-basin units goes through severe water scarcity for 3–12 months in the year. These sub-basins include nearly a billion people. Generally, the results of this study provide insights regarding the number of people and country-basin units experiencing low, moderate, significant and severe water scarcity at a monthly temporal resolution. These insights might help these basins’ sharing countries to design and implement sustainable water management and sharing schemes.

## Introduction

Water is one of the most important renewable natural capitals that drives life. In nature this resource is allocated unevenly in different forms. Around 97.5% of the global water exists as salt water and 2.5% as freshwater. Out of the total freshwater only 31.1% is available in aquifers, lakes and rivers for human consumption. The rest is trapped in the ice caps^1,2^. Moreover, this available fresh water is spatially and temporally unevenly distributed^3–7^.

In its global risk report the World Economic Forum^8^ listed water crisis as fourth & ninth in terms of impact and likelihood respectively. In recent years due to the rising water demand for agricultural, industrial and domestic purposes the demand for fresh water supply has been growing at an alarming rate^9–13^. This demand for water like the available water varies through space and time as well^6,7^. Hence, understanding the temporal and spatial variation of water scarcity is important to identify areas where the volume available water and water footprint are unmatched at certain period of time. This information could be useful for designing sustainable fresh water management policies. In order to give a global picture of the problem by identifying areas facing water scarcity at different time periods various studies have been conducted at different spatial and temporal resolutions. In addition, these studies also differ in terms of metrics of water use and how ecological water requirements are taken into account.

Falkenmark *et al*.^14^ classified water scarcity as population driven and demand driven. These two classifications of water scarcity define water use differently. Population driven water scarcity distribute the available water among the population residing within a spatial unit^14,15^. Kummu *et al*.^16^ conducted global assessment of population driven water scarcity at sub-basin spatial and annual temporal resolutions. Whereas Arnell^17,18^ did global assessment of water scarcity at 5 × 5°spatial resolution taking population size as the measure of water use. They used macro-scale hydrological model to estimate current and future water resource availability and aggregated the results to watershed scale. The other metrics of water scarcity is demand driven. This measure takes water withdrawn or consumed as measure water use^12,14,19,20^. Water scarcity studies by Oki and Kanae^1^; Vorosmarty *et al*.^7^; Oki *et al*.^16^ and Wada *et al*.^17^ assessed water scarcity at 30 arc-minute resolution annually for the target year 1995 taking available water as natural runoff minus upstream water consumption and water withdrawal as a measure of water use. In addition, Alcamo *et al*.^19^ and Alcamo & Henrichs^21^ also examined the annual water scarcity at basin level by setting natural runoff and water withdrawn as available water and water footprint respectively. While Hoekstra *et al*.^7^ and Mekonnen & Hoekstra^22^ determined monthly water scarcity at basin and 30 arc-minute spatial resolutions respectively by defining water footprint as water consumed rather than withdrawn.

The other difference among global water scarcity studies is temporal resolution. Some researchers conducted the global assessment of water scarcity at an annual time step^1,23,24^. However, water scarcity might not be revealed throughout the year^6,25–27^. This is mainly due to the fact that available water and water footprint fluctuates monthly and seasonally^6,7^. Hence, annual assessment might not be sufficient enough to obtain insights in to intra-annual water scarcity variations^26–28^. Therefore, the assessment of water scarcity at monthly temporal resolution is crucial. Assessment of water availability and footprint at a monthly time step provides insights into water scarcity which are not revealed by assessment at an annual temporal resolution. Taking this in to consideration Hoekstra *et al*.^7^; Alcamo & Henrichs^21^; Wada *et al*.^25^; Van Beek *et al*.^28^; Smakhtin *et al*.^29^ and Mekonnen & Hoekstra^30^ determined monthly water scarcity at 30 arc-minute and 60 arc-minute resolutions taking in to account the accumulated runoff and only the amount of water consumed not withdrawn as available water and water footprint.

Spatial resolution of water availability and footprint assessment determines the extent to which water scarcity variations are observed among spatial units. Hoekstra *et al*.^7^; Alcamo *et al*.^19^ and Alcamo & Henrichs^21^ calculated monthly water scarcity at basin level aiming to provide water stress insights at a spatial resolution familiar to water resource managers. But their study did not capture spatial variations of water footprint and availability within river basins. Oki & Kanae^1^; Wada *et al*.^25^ *and* Smakhtin *et al*.^29^did water scarcity assessments at higher spatial resolutions but they failed to take in to account environmental water needs. Following their work Mekonnen & Hoekstra^30^ calculated the water scarcity at 30 arc-minute resolution on a monthly time step considering upstream water consumption and environmental water needs. But the insights obtained at this spatial resolution might be difficult for water resource management experts and policy makers to use because it is not a familiar spatial resolution among them^7,31^. Hence, water scarcity studies at least at sub-basin level are needed to make water scarcity assessment results convenient for use by water policy makers. Understanding variations among sub-basins in terms of water footprint and availability are even more crucial particularly when assessing water scarcity in border crossing river and lake basins. Among the recent efforts to capture water scarcity at this spatial resolution within transboundary fresh water resources are studies by Wada & Heinrich^32^ and Munia *et al*.^33^.

The results of water scarcity assessments also differ depending on how environmental water needs are internalized. The under estimation or over estimation of water scarcity might depend not only on the way water footprint is measured but also on how much of the accumulated flow is left to sustain environmental services. For instance Hoekstra *et al*.^7^ and Mekonnen and Hoekstra^30^ assigned 80% of the natural runoff within the basins as precautionary approach based on the study by Richter *et al*.^11^ while Munia *et al*.^33^ assigned 30–40% of the natural discharge for fulfilling ecological water requirements. On the other hand, Smakhtin *et al*.^29^ suggested that approximately 20–50% of the mean annual river flow within basins needs to be allocated to freshwater dependent ecosystems to maintain their ecological integrity.

In this research we build up on the previous studies by undertaking our water scarcity assessment under the following settings. First, our assessment is solely focused on transboundary river and lake basins because these basins span 151 countries, include around 42% of the world’s population, cover 42% of the total land area of the Earth and produce roughly 54% of the global river discharge^34–36^. For these reasons water scarcity in these river basins could result international water conflicts which can have serious socio-economic and environmental consequences. Furthermore, water stress in transboundary water resources might also be an indication of water shortage within non-transboundary freshwater basins. Most of the time countries opt to compete for internationally contested water resources when their internal water resources are water scarce. Second, we conducted our assessments at spatial resolution of country-basin unit because these sub-basins are managed by different water resource management policies from multiple riparian countries. Besides the knowledge of water stress in transboundary river and lake basins at this spatial resolution is limited^33^. The impact of water use at this spatial resolution has been studied globally only by Wada & Heinrich^32^ and Munia *et al*.^33^. Thirdly, country-basin unit to country-basin unit flow accumulation has advantage over the conventional pixel to pixel and basin wide water availability and footprint determination for the following four main reasons. (1) Every riparian country of a transboundary basin claims the basin’s water within its borders based on the principle of “absolute territorial sovereignty”. Country-basin units of transboundary basins are within the sovereign boarders of their different riparian countries. Hence this spatial resolution is convenient for policy makers to design and implement practical water management policies within the basins. (2) At country-basin unit to country-basin unit spatial resolution the water within the sub-basin is available for use throughout the sub-basin unlike pixel to pixel spatial resolution^31^.(3) Water scarcity assessment at country-basin unit spatial resolution enable us to capture variations in terms of water footprint and availability within transboundary basins. Insights on water availability and water footprint in these sub-basins are very important for water allocation negotiations within border crossing basins. (4) The only water scarcity assessment study in global transboundary river and lake basins at country-basin unit spatial resolution is by Munia *et al*.^33^. Their study is done at annual temporal resolution. However the degree of water scarcity varies within a year^7,27,37^. In this work we conducted demand driven water scarcity assessment at this spatial resolution and monthly time step. To our knowledge there is no global water scarcity assessment done at these spatial and monthly temporal resolutions yet.

## Methods

**Figure 1:**
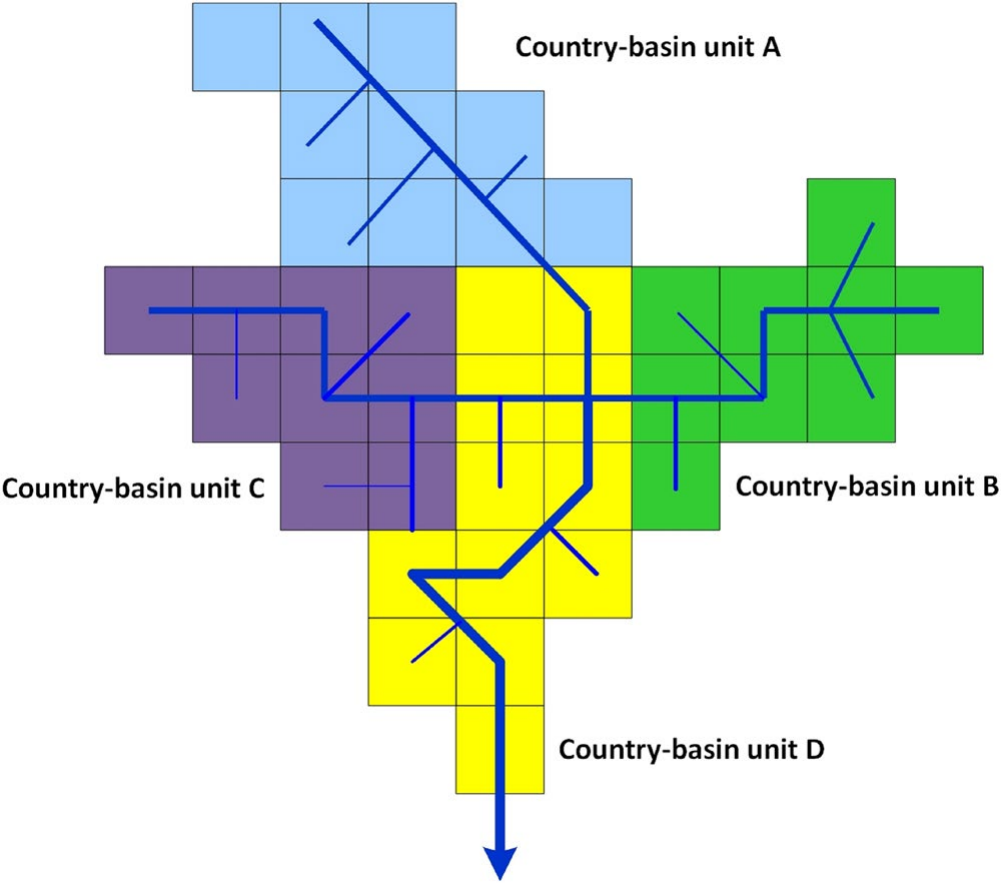
Country-basin units of a typical transboundary basin.

In this research mapping monthly blue water scarcity in global transboundary river and lake basins at country-basin spatial and monthly temporal resolutions was conducted. The map of country-basin units was obtained by meshing raster maps of country and basin boundaries of the same resolution. Raster maps of country and basin boundaries were obtained by converting country and basin polygon maps from Transboundary Waters Assessment Program Data Portal^36^ to 30 arc-minute resolution. The schematic illustration of country-basin units is shown in Fig. 1.

The monthly water footprint per country-basin unit was calculated by aggregating the global water footprint obtained from Mekonnen & Hoekstra^38^ at 30 arc-minute resolution to a country-basin spatial resolution. These water footprints were calculated by summing agricultural, industrial and domestic water consumptions. They are mean values for the study period. When determining the agricultural water footprint Mekonnen & Hoekstra^38^ used two water soil balance scenarios. Then blue water consumption was calculated as the evapotranspiration during the growing period. They determined the domestic and industrial water footprints by distributing the national water footprint data from the Food and Agricultural Organization of the United Nations (FAO)^39^ according to the population density from Center for International Earth Science Information Network (CIESIN)^40^. In areas where the runoff is completely depleted ground water is assumed to be the source of water.

**Figure 2:**
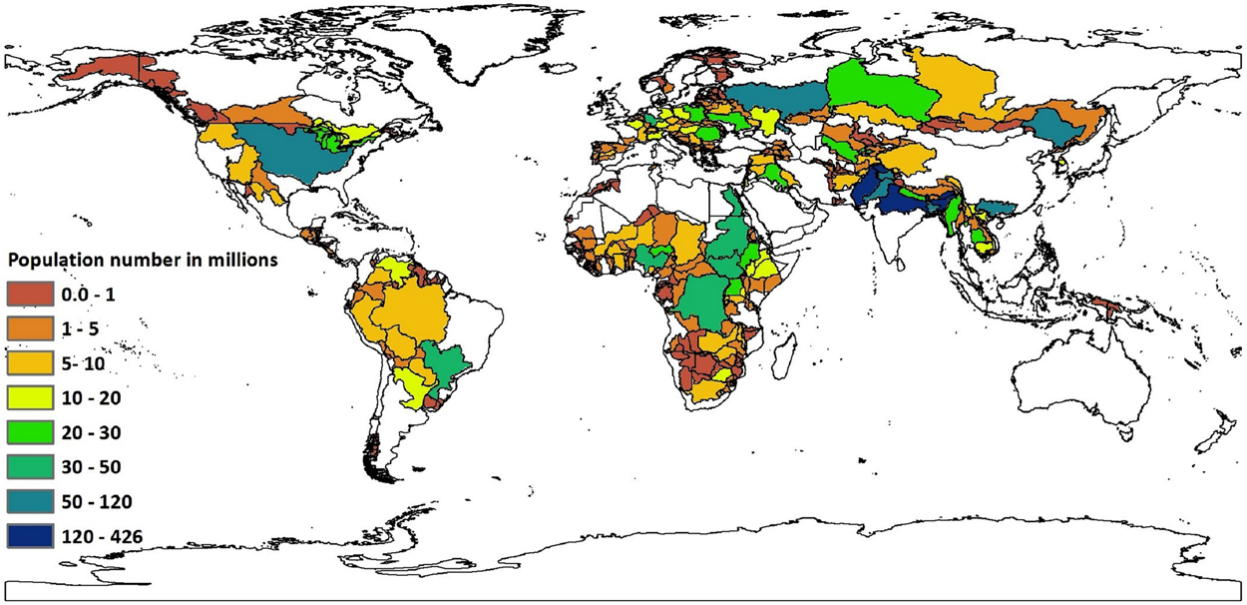
UN-Adjusted population count per country-basin unit of transboundary river basins in millions for the year 2000. Population count within each country-basin unit was obtained by aggregating the UN adjusted population count for the target year from Center for International Earth Science Information Network (CIESIN)^42^ to a country-basin spatial resolution. This map was generated with ArcGIS 10.2 for desktop (http://www.esri.com/sofware/arcgis).

The monthly available water per country-basin unit after upstream water consumption was determined by adding the accumulated actual runoff and the water footprint within each country-basin unit. Actual runoff is equal to natural runoff minus upstream water consumption. In order to determine the monthly accumulated actual runoff within each country-basin unit we used flow direction data from Fekete *et al*.^41^. Therefore, country-basin units which are greater than 10000 km^2^ where represented, those above 25000 km^2^ more reliably. The actual runoff data was also acquired from Fekete *et al*.^41^. The monthly accumulated actual runoff was obtained by multiplying the actual runoff by accumulated area. The environmental water needs were taken into account on the basis of previous studies by Hoekstra *et al*.^7^; Richter *et al*.^11^ and Mekonnen & Hoekstra^30^.Therefore, we setup environmental water requirements to be 80% of the water available after upstream consumption within each country-basin unit of each transboundary river basin. As stated by Richter *et al*.^11^ and Mekonnen & Hoekstra^30^ this is precautionary water amount needed for maintaining the integrity of ecological health within the sub-basins. UN-Adjusted population count which is estimate of the number of persons per 30 arc-second (~1 km) grid cell for the target year 2000 was obtained from Center for International Earth Science Information Network (CIESIN)^42^ and was resampled to 30 arc-minute resolution. Population number per country-basin unit is shown in Fig. 2.

The water scarcity level is classified as low, moderate, significant and severe according to Hoekstra *et al*.^7^. This classification is shown in Table 1.

**Table 1:** Levels of blue water scarcity.

Water Scarcity Value	Water Scarcity Level	Remark
<100%	Low	The water allotted for sustaining ecological services is untouched
100–150	Moderate	The water assigned for preserving ecological services is slightly not met
150–200	Significant	The water allocated for conserving ecological services is violated considerably
>200	Severe	The water apportioned for maintaining ecological services is significantly disturbed

### Data Availability

All data needed to evaluate the conclusions in the paper are present in the paper and/or the Supplementary Materials. Additional data related to this paper can be requested from the authors.

## Results and Discussion

In recent years water scarcity studies at different temporal and spatial scales have been conducted in order to understand extent of the problem. Hoekstra *et al*.^7^ conducted monthly water scarcity assessment for the major river basins of the world and identified river basins facing low to severe water scarcity. Following their study Mekonnen and Hoekstra^30^ also determined the global water scarcity at monthly temporal resolution and higher spatial resolution. These two recent studies provided important scientific information about the level of monthly blue water scarcity globally taking into account environmental water requirements and only the water consumed as water footprint. But if we entirely focus our attention on understanding water scarcity within transboundary river basins for the reasons mentioned in the introductory section of this paper these two studies have the following limitations. Hoekstra *et al*.^7^ chose to conduct their study at the river basin spatial scale aiming to provide scientific insights that can be easy used by water management experts. But their study might under or overestimate the level of water scarcity within country-basin units and the number of people facing water scarcity because they assumed that the rivers’ natural runoff is available for consumption throughout the basins. This is not the case in reality especially within transboundary river basins because the different sections of the basins lie within the political borders of their riparian countries. Only the water that emanates within the sub-basins plus the accumulated runoff after upstream consumption is available for the middle stream and downstream sub-basins of the rivers. Furthermore, the majority the rivers’ natural runoff might not always emanate from the upstream section of the basins. This also limits the availability of water to the upstream sub-basins. Therefore, this spatial resolution is too coarse to take in to account the water availability and consumption variations among the sub-basins of the rivers located within their riparian countries’ boarders. On the other hand the study by Mekonnen and Hoekstra^30^ was done at a spatial resolution of 30 arc minute. This study also has similar drawback. Because the study was conducted at a very high spatial resolution, it limits the available water and water footprint at a pixel spatial resolution. As the result the study did not consider the fact that the water available within each pixel could be accessible for consumption by people living within adjacent pixels too. In addition this spatial resolution is not convenient for use by water management experts^7^ and not coarse enough to take in to account the border crossing nature of transboundary river basins. Recognizing these limitations Munia *et al*.^33^ conducted annual water scarcity study within global transboundary river basins at a country-basin unit spatial resolution. They found that water stress affects 0.95–1.44 billion people or 33–51% of the population in transboundary river and lake basins when upstream consumption was not taken in to account. The water stress level for 30–65 sub-basins increased after they take upstream water footprint into consideration. These sub-basins include 0.29–1.13 billion people. But because their study was done at annual temporal resolution monthly and seasonal water scarcity variations among these spatial units were not made visible by the study.

As discussed above the previous studies on water availability and footprint presented water scarcity assessments within river basins at temporal and spatial resolutions that are either too low or too high to understand the variations within transboundary river basins. This research article aims to fill these research gaps. Thus, it presented monthly assessment of water scarcity in global transboundary river and lake basins at spatial resolution of country-basin units. Moreover, our assessment differs from the previous studies by Hoekstra *et al*.^7^; Richter *et al*.^11^; Mekonnen and Hoekstra^30^; Munia *et al*.^33^ because we assumed that only 20% of the accumulated flow within each country-basin unit is available for human consumption. The rest is assumed to be allocated for satisfying environmental water requirements. This assumption respects riparian countries’ rights to use the basins’ water within their sovereign boarders. The maps and data of available water per country-basin unit for each month of the year are shown as Supplementary Fig. S2 and Table S1.

The results obtained in this assessment have showed monthly variations in terms of water scarcity among the 560 country-basin units of the 248 transboundary river and lake basins investigated in this study. In our investigation we found that around 1.6 billion people living within the 328 out of the 560 country-basin units of transboundary river and lake basins considered in this study face severe water scarcity at least for one month of the year. This is equal to 63.52% of the total population within transboundary river and lake basins, 90.15% of the total population facing water scarcity at least one month of the year and 59.26% of the 2.7 billion people who are reported to be living under severe water scarcity for at least one month of the year within the 405 major river basins according to the study by Hoekstra *et al*.^7^. Whereas 175 country-basin units goes through severe water scarcity for 3–12 months. These sub-basins include nearly a billion people out of the total population within border crossing basins. Therefore, nearly a billion people experience severe water scarcity at least for three months within the year. On the other hand, 13 country-basin units suffers from severe water scarcity every month of the year. These are Awash in Somalia & Djibouti, Gash in Sudan, Baraka in Eritrea & Sudan, Atui in Mauritania & Western Sahara, Kogilni in Moldova, Lake Chad in Libya, Nile in Eritrea & Egypt and Niger in Algeria. The number of people and number of country-basin units facing low, moderate, significant and severe water scarcity for n months per year are shown in Table 2.

**Table 2:** Number of people (in millions) and number of country-basin units facing low water scarcity (<100%), moderate water scarcity (100–150%), significant water scarcity (150–200%) and severe water scarcity (>200%) for n months per year. Period: 1996–2005. UN adjusted population count for the target year 2000 from Center for International Earth Science Information Network (CIESIN)^39^ was used.

Number of months per year (n)	Number of people (in millions) facing low, moderate, significant and severe water scarcity during n months per year				Number of country-basin units facing low, moderate, significant and severe water scarcity during n months per year			
	Low water scarcity	Moderate water scarcity	Significant water scarcity	Severe water scarcity	Low water scarcity	Moderate water scarcity	Significant water scarcity	Severe water scarcity
0	44.35	1,483.91	1,738.13	926.41	12	465	485	233
1	166.81	336.55	602.71	257.14	32	60	48	89
2	94.19	176.54	149.03	230.22	5	28	21	64
3	44.29	520.70	14.99	563.34	11	7	3	38
4	58.37	0	12.28	101.62	12	0	2	51
5	454.10	0	0	28.52	7	0	0	18
6	27.35	0	0	43.07	17	0	0	9
7	55.22	0	0.56	125.11	23	0	1	6
8	68.76	0	0	158.36	40	0	0	9
9	176.56	0	0	4.19	45	0	0	4
10	354.55	0	0	0	69	0	0	3
11	217.96	0	0	34.72	83	0	0	24
12	755.19	0	0	44.39	204	0	0	12
Sum	2,517.7	2,517.7	2,517.7	2,517.7	560	560	560	560

**Figure 3:**
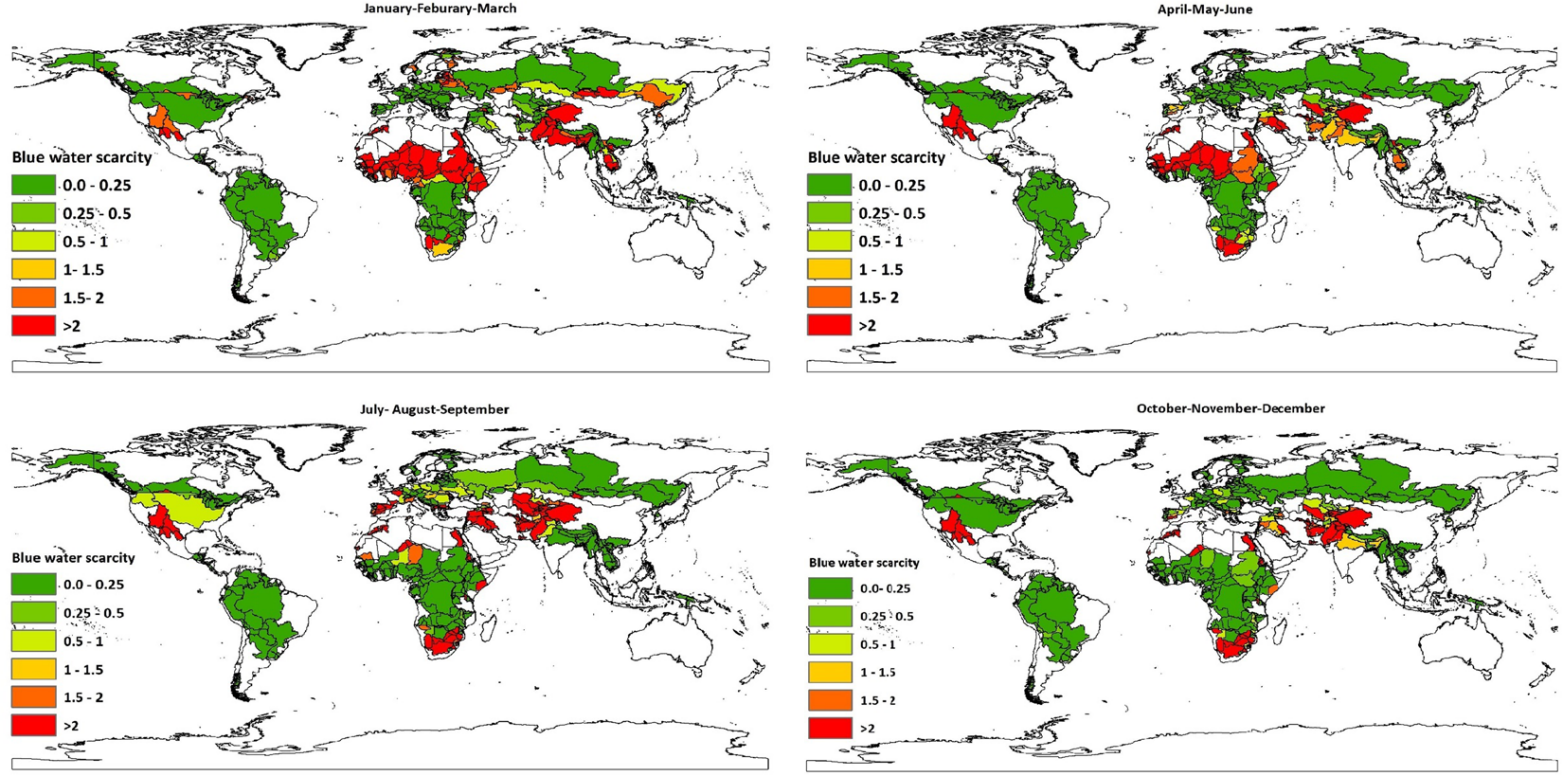
Quarterly averaged monthly blue water scarcity at country-basin unit spatial resolution. Period: 1996–2005. Blue water scarcity at country-basin mesh spatial resolution is defined as the ratio of the blue water footprint to the available blue water within each sub-basin. These maps were generated with ArcGIS 10.2 for desktop (http://www.esri.com/sofware/arcgis).

Seasonal assessment of water scarcity shows that the number of people and sub-basins living under low, moderate, significant and severe water scarcity during the four seasons are different. From January to March among the 560 country-basin units we assessed for water scarcity 7,118 and 134 are under moderate, significant and severe water scarcity respectively. While the remaining 301 experience low water scarcity. Therefore, within the 259 country-basin units carrying 1.37 billion people ecological water needs are disturbed. Out of this number 82.96% go through severe water scarcity. Most of these people live below the Sahara Desert and above the equatorial region of Africa and in south Asia. Moderate to severe water scarcity affects around a billion people from April to June. Out of this number 26.2% endures severe water scarcity while 18.8% experience significant water scarcity. The remaining 54.95% spends this season under moderate water scarcity. These people inhabit 173 country-basin units. The number of people living under severe water scarcity is lowest during this time of the year. This could be due to the fact that country-basin units of Indus and Ganges-Brahmaputra-Meghna river basins which are characterized by high population number are under low to significant water scarcity during this period. From July to September 0.47 billion people in 132 country-basin units live under moderate to severe water scarcity. Approximately 84.55% of the population endure severe water scarcity. Globally the number of people dwelling within the transboundary river basins’ country-basin units where the assumed ecological water requirements are violated is the lowest during this season. This might be because country-basin units of the Ganges-Brahmaputra-Meghna basin which includes around half a billion people in India alone are under low water scarcity. From October to December 99 country-basin units go through moderate to severe water scarcity. Around 0.88 billion people populate these country-basin units. Nearly 0.38 billion people living within 74 of the 99 country-basin units are under severe water scarcity. Maps that depict quarterly averaged monthly blue water scarcity at country-basin unit spatial resolution are shown in Fig. 3.

There are 37 country-basin units which undergo severe water scarcity throughout the four seasons. Among these country-basin units the main ones are Colorado, Rio Grande, Yaqui country-basin units in Mexico & the United States, Nile river basin in Egypt & Eritrea, Orange river in Namibia & Botswana, Tigris-Euphrates/Shatt al Arab in Jordan, Juba-Shibeli in Somalia, Niger river’s & Lake Chad’s country-basin units in Algeria, Tarim in China, Helmand in Afghanistan and Indus in Pakistan. Tables 3 and 4 depict number of people and number of country-basin units facing low, moderate, significant and severe water scarcity per season respectively.

**Table 3:** Number of people (in millions) facing low water scarcity (<100%), moderate water scarcity (100–150%), significant water scarcity (150–200%) and severe water scarcity (>200%) per season. Period: 1996–2005. UN adjusted population count for the target year 2000 from Center for International Earth Science Information Network (CIESIN)^39^ was used.

Season	Number of people (in millions) facing low, moderate, significant and severe water scarcity per season			
	Low water scarcity	Moderate water scarcity	Significant water scarcity	Severe water Scarcity
January-February-March	1,147.32	11.67	222.09	1,137.64
April-May-June	1,494.52	562.86	192.83	268.51
July-August-September	2,047.77	23.08	50.47	397.40
October-November-December	1,638.08	454.17	43.19	383.28

**Table 4:** Number of country-basin units facing low water scarcity (<100%), moderate water scarcity (100–150), significant water scarcity (150–200%) and severe water scarcity (>200%) per season. Period: 1996–2005.

Season	Number country-basin units facing low, moderate, significant and severe water scarcity per season			
	Low water scarcity	Moderate water scarcity	Significant water scarcity	Severe water scarcity
January-February-March	301	7	118	134
April-May-June	387	13	45	115
July-August-September	428	8	20	104
October-November-December	461	11	14	74

**Figure 4:**
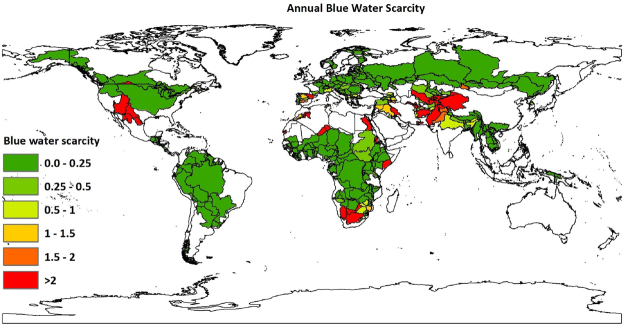
Average annual blue water scarcity per country-basin unit. Period: 1996–2005. Blue water scarcity at country-basin mesh spatial resolution is defined as the ratio of the blue water footprint to the available blue water within each sub-basin. This map was generated with ArcGIS 10.2 for desktop (http://www.esri.com/sofware/arcgis).

The authors also conducted annual water scarcity study to compare the results with those done at monthly temporal resolution. The annual water footprint was obtained by summing the ten year average water footprint for each month of the year^7,43^. The sum of annual water footprint^7^ and annual runoff^41^ accumulated within each country-basin unit after upstream consumption was taken as annual available water. Our annual assessment of the problem showed that nearly half a billion people experience water scarcity. Out of this number nearly 34.37% suffers from severe water scarcity. Therefore, the results obtained at annual temporal resolution considerably underestimates the number of people and country-basin units suffering from water scarcity. Figure 4 shows the annual water scarcity per country-basin unit.

Generally, water scarcity within country-basin units of transboundary basins is due to low water availability and large water footprint. But the main reason for water scarcity within most of the country-basins units of border crossing rivers is temporal and spatial mismatch of available water & water footprint. For the midstream & downstream country-basin units large blue water footprint in the upstream sub-basins of the river and lake basins is additional reason for water scarcity.

Limpopo river’s sub-basins in southern Africa and Ganges-Brahmaputra-Meghna’s sub-basins in India & Bangladesh are examples of country-basin units which suffers from water scarcity because the time periods for large available water and water footprint do not coincide^30^. Water scarcity in country-basin units could also be due to huge water footprint. Country-Basin units of the Colorado river basin & Rio Grande sub-basins in United States & Mexico and lower Nile in Egypt are the notable country-basin units of transboundary river basins which experience water scarcity as the result of their large water footprints^19,21,44,45^. The other reason for water scarcity in country-basin units is upstream water consumption. The country-basin units of Aral Sea and Lake Chad are amongst the transboundary fresh water resources which endure water scarcity as a result of upstream water consumption^46–49^. The Senegal river’s sub-basin in Mauritania, Lake Chad’s & Niger river’s sub-basins in Algeria, Tagus/Tejo & Guadiana country-basin units in Spain and Tarim river basin in China, which drains Taklamakan Desert, are examples of transboundary basins’ sub-basins which goes through water scarcity because they are located in arid regions(low runoff and high temperature)^21^. High population count or/and density in addition to aridity is the cause for water stress within country-basin units of Lake Chad & Niger river in Algeria, Tigris-Euphrates/Shatt al Arab river’s country-basin units, lower Nile in Egypt and Juba-Shibeli river in Ethiopia & Somalia. On the other hand within the country-basin units of river basins like the Volga, Congo and Amazon water is not used intensively^19^. As the result water scarcity is not a problem within the country-basin units of these river basins throughout the four seasons.

The runoff estimates from Fekete *et al*.^41^ have uncertainty of 5% while the runoff measurements from the Global Runoff Data Centre (GRDC)^50^ against which these values are calibrated have uncertainties of ±10–20%^41^. In addition, the blue water footprint estimates from Mekonnen & Hoekstra^38^ have ±20% uncertainty. Therefore, we conducted sensitivity testes by varying the water footprint, the available water and the water assigned for maintaining ecosystem services within each sub-basin. The water scarcity values in this article are calculated under the assumption that only 20% of the total water within each country-basin unit is allocated for human consumption. If we increased this to 40% the number of people facing severe water scarcity at least one month of the year reduces from 1.59 to 1.21 billion. When the available water is in ±20% range the number of people facing severe water scarcity at least one month of the year is between 1.59 and 1.61 billion people. On the other hand, increasing the available water within the country-basin units by 50% reduces the number of people facing water scarcity from 1.59 to 1.51 billion while decreasing it by the same amount increases the number from 1.59 to 1.77 billion. Changing water footprint of each country-basin unit in the ±20% and ±50% ranges results 1.55–1.61 and 1.21–1.62 billion people facing severe water scarcity at least for a month within the year respectively. Therefore, the results of the water scarcity assessment are hardly sensitivity to changes in water footprint and availability as well as to the amount of water assigned for sustaining ecological services within the country-basin units. This might be because time periods of large water consumption and availability do not coincide^5–7^. The water scarcity values for smaller country-basin units are more sensitive to these changes than those of large country-basin units^7^.

Even though the results of this research depicted the state of monthly blue water scarcity within transboundary basins’ country-basin units. The following aspects should be taken in to account while interpreting the results because the data used have the following drawbacks. First, the role reservoirs have in terms of increasing the available water is not investigated in detail in this research. Second, the water footprint data from Mekonnen & Hoekstra^38^ do not take in to account loss of water in arid regions to evaporation and inter-basin water transfers. In addition the monthly water footprint and runoff used are for an average year thus hides inter-annual variability^38^.

In this study the authors conducted monthly water scarcity assessment within the country-basin units of the main transboundary river and lake basins of the globe. Future studies can build on this research and address the following main research gaps. First, reservoirs are known to play a huge role in terms of mitigating water scarcity. Hence, reservoirs role^51^ in terms of mitigating water scarcity should be explored. Second, only blue water availability and footprint were taken in to account in this study. Future studies should take in to account green water availability and footprint in their water scarcity assessments^5,22,42^. Third, ecological water requirements should be studied further in detail within the transboundary river and lake basins^7,22^. Fourth, in this research the measure of water footprint is water consumption. This underestimates the water footprint because the quality of the water withdrawn but return back to the water bodies was not taken in to account^22,33^. Fifth, water availability and footprint within transboundary basins has been changing together with the continuously changing socio-economic, demographic and environmental conditions. Hence this assessment needs to be updated. Furthermore, our investigation did not take into account country water rights, international and regional agreements. Future researches take these in to account besides environmental flow in order to give full picture for policy and decision makers. Additionally, further studies predicting future water scarcity scenarios under the impact of climate change^9,48,49,52^ are also essential to design sustainable water management and sharing systems for transboundary river and lake basins.

## Conclusion

The sustainable management of the unevenly distributed fresh water resources holds the key to alleviate water scarcity. Transboundary basins are among these important fresh water resources facing this problem. These river and lake basins account for large percentage of the global runoff, covers large area and include huge portion of the world’s population. Besides, their border crossing nature makes them niches for international water conflict or cooperation. Therefore, managing these river basins in a sustainable way not only reduces the number of people facing water scarcity but also avoids international water conflicts. In addition, it also creates opportunity for basin wide protection of these basins’ ecosystems. For these reasons the assessment of water footprint and availability within these river basins at country-basin spatial resolution and monthly time step is essential.

In this study we investigated demand driven water scarcity within transboundary basins at country-basin unit spatial and monthly temporal resolutions. The monthly water footprint per country-basin unit was calculated by aggregating the global water footprint to a country-basin spatial resolution. These water footprints were calculated by summing agricultural, industrial and domestic water consumptions. The monthly available water by country-basin unit after upstream water consumption was determined by adding the accumulated actual runoff and the water footprint within each country-basin unit. The monthly accumulated actual runoff with in each country-basin unit was obtained by multiplying the actual runoff by accumulated area. This way we were able to capture the monthly variations in terms of water availability and footprint among these spatial units. The results of this assessment identified transboundary basins’ country-basin units experiencing low, moderate, significant and severe water scarcity. These results have the following practical implications on how water is managed within transboundary river basins. (1) Identifying the country-basin units were water demand and availability are mismatched through time and space helps water allocation experts to add the spatial and temporal variations of water availability and footprint within the basins in to their water management systems. (2) The results take in to account upstream water consumption and the amount of water that emanates from the downstream country-basin units of the basins that is not available for consumption in the upstream country-basin units. Thus, they can be useful inputs for water managers to design water sharing schemes which are fair and practical. Furthermore, the number of people living under these different levels of water scarcity at a monthly time step is also determined. In a nut shell our results showed that around 1.6 billion people living within the 328 country-basin units out of the 560 we assessed in this study endures severe water scarcity at least one month of the year. Out of this number nearly a billion people in 175 country-basin units goes through severe water scarcity at least for three months within the year. These insights could be as base for further researches at these spatial and temporal resolutions that can assist policy makers and water managers from riparian countries of transboundary basins in their efforts to manage these basins’ water capital in a way that is fair, efficient and sustainable.

## Electronic supplementary material

Dataset 1

Supplementary Results

Dataset 4

Dataset 3

Dataset 2
