# Supplementary material for: Mapping Monthly Water Scarcity in Global Transboundary Basins at Country-Basin Mesh Based Spatial Resolution
Source: Sci Rep. 2018 Feb 1;8:2144. doi: 10.1038/s41598-018-20032-w (PMC5794897; doi:10.1038/s41598-018-20032-w)
Supplement: Supplementary file 2 — Supplementary Results [file 41598_2018_20032_MOESM2_ESM.doc]

# **Mapping Monthly Water Scarcity in Global Transboundary Basins at Country-Basin Mesh Based Spatial Resolution**

**Dagmawi Mulugeta Degefu*****, Weijun He**^+^**, Zaiyi Liao**^+^**, Liang Yuan, Zheng Wei Huang, Min An**

# **Supplementary Results**

**Monthly Blue Water Footprint by Country-Basin Spatial Unit**

The water footprint for the country-basin units of transboundary rivers was obtained by aggregating the monthly data from Mekonnen & Hoekstra^35^.This data is the sum of the water footprints from the three main water consuming sectors (agricultural sector, industrial sector and domestic sector) at 30 arc-minute resolution. Some of the river basins showed all year-round uniform water footprint throughout their country-basin units, while others experience monthly variation in water footprint among their sub-basins which are located within the sovereign boarders of their riparian countries. Monthly maps of water footprint are presented in **Fig.S1** while the water footprint values per country-basin unit are given in **Table S2**.

In Africa the water footprints of country-basin units of the Nile river basin in Sudan and Egypt are high all year round. This is mostly due to large agricultural, industrial and domestic water consumption in these sub-basins of the river. The water footprint of the river’s sub-basins in Ethiopia and equatorial region is lower than the water footprints of the sub-basins in Sudan and Egypt. This could be due to the non-consumptive nature of water requiring economic activities and abundance of internal water resources in the country-basin sub-basins of the river in these riparian countries. The country-basin units of the Orange and Limpopo river basins in southern Africa also shows large blue water footprint for one or two months of the year compared to the rest of Africa’s transboundary river’s country-basin units except for the Nile’s.

In Asia the Ganges-Brahmaputra-Meghna in India and Indus in India & Pakistan have very huge water footprints all year around, mainly due to high population density and/or count and all year-round water intensive agricultural practice. While this river’s sub-basins in China, Nepal and Bangladesh have small water footprint relative to the other country-basin sub-basins. Tarim Rivers’ country-basin unit in China and Aral Sea’s country-basin units in Uzbekistan and Kazakhstan also show large water footprints. In Europe the Volga River’s country-basin unit in Russia experience very high water footprint for four to five months with in a year. The Donx River’s sub-basin in Russia, Dnieper sub-basin in Ukraine and Danube’s sub-basin in Romania also show high water footprint relative to their country-basin units in the other riparian countries.

In North America the country-basin units of the Mississippi, Colombia and Colorado River basins in the United States have large water footprint most of the year. The water footprint of St. Lawrence and Nelson-Saskatchewan rivers’ country-basin units in the United States and Canada as well as Rio Grande in Mexico have very large blue water footprint but less than the water footprint of Mississippi, Colombia, Colorado, Yaqui and Rio-Grande country-basin units in the United States.

In South America the country-basin units of the La Plata river basin in Argentina and Brazil as well as Amazon River’s country-basin unit in Ecuador shows large water footprint for three to four months of the year compared to the water footprints of their sub-basins in the other riparian countries.

Generally, in most of these rivers the level of water footprint does not necessarily indicate low, moderate, significant or severe water scarcity. This is because as much as water scarcity depend on spatially and temporally variable water footprint it also depends on the water availability, which is equally temporally and spatially variable.


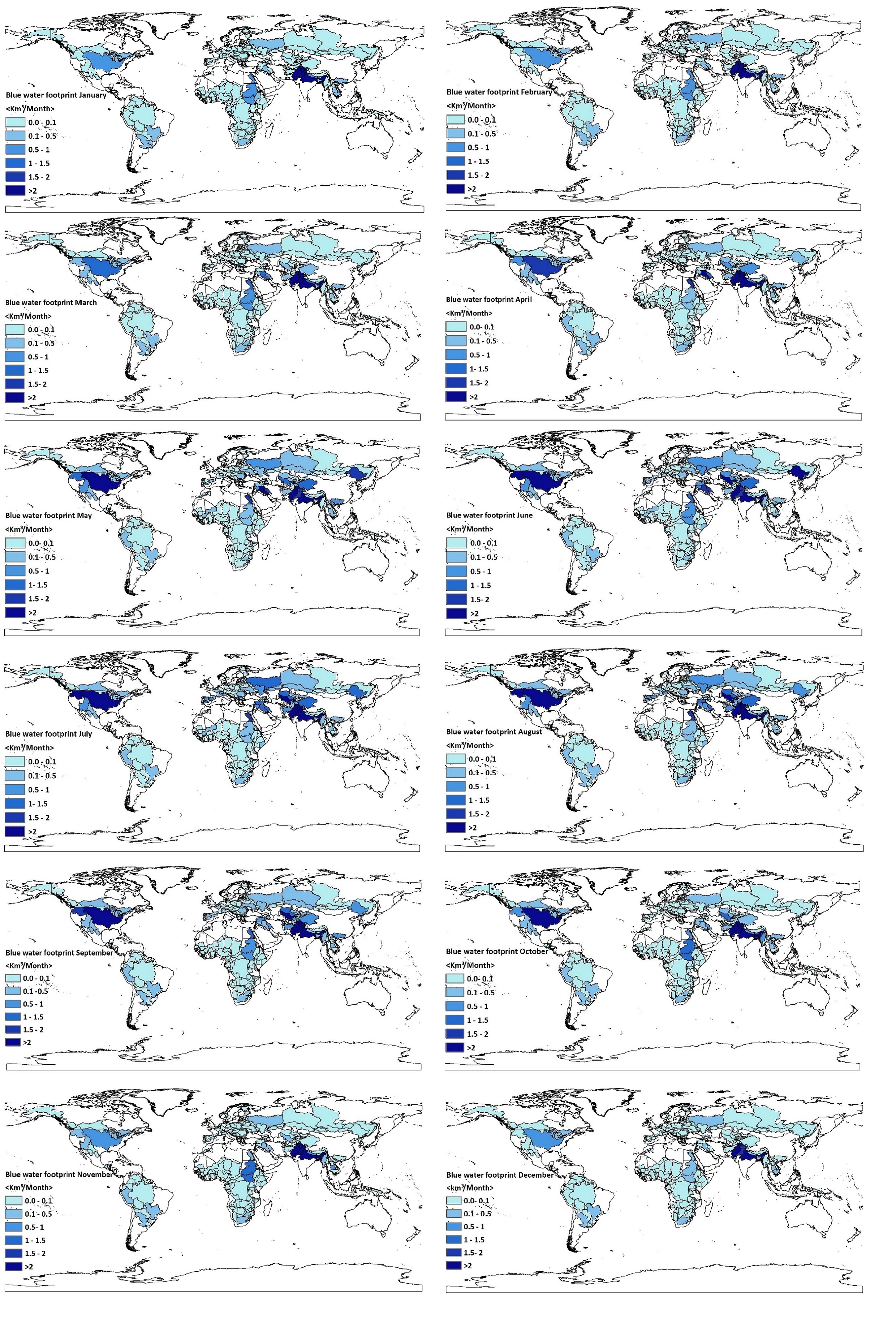


**Figure S1.** Monthly Blue water footprint per country-basin unit of transboundary basins in km^3^/month. Period: 1996–2005. The blue water footprint per country-basin unit was calculated by aggregating the global water footprint obtained from Mekonnen & Hoekstra^38^ at 30 arc-minute resolution to country-basin spatial resolution. Maps generated with ArcGIS 10.2 for desktop (<http://www.esri.com/sofware/arcgis>).

**Monthly Available Water by Country-Basin Spatial Unit**

In this section the water available for consumption in transboundary river and lake basins’ country-basin units is presented considering upstream water consumption and environmental water needs. The available water is calculated in a way different from the previous studies by Hoekstra *et al.*^5^; Mekonnen & Hoekstra^28^; Richter *et* *al.*^8^; Munia *et al.*^31^.We presumed that 20 % of the accumulated flow within each country-basin unit of each transboundary basin as the available water for consumption without compromising environmental water requirement. This assumption respects the rights of transboundary river and lake basins’ riparian countries to use the basins’ water within their sovereign boarders according the principle of “absolute territorial sovereignty”. The maps showing the available water per country-basin unit during each month of the year are shown below in **Fig. S2** and data can be found in **Table S3** of the supplementary material.

Most of the country-basin units of equatorial Africa’s transboundary rivers like Congo, Ogooue, Utamboni, Mbe, Nyanga and Benito/Ntem have uniform and high water availability, because runoff in the region is high for long duration within a year. Sanaga sub-basin in Cameron, Congo River in Cameron & African Republic and Ogooue in Gabon are the exceptions which have low available water compared to the country-basin units in the other riparian countries. This might be due to high upstream water consumption, low water availability and/or tributary rivers discharge difference within the sub-basins. The country-basin units of the Zambezi river basin have abundant water from December to May but decreases during the months after. The Nile river basin is one of the most important river basin in equatorial, eastern and northern Africa. The country-basin units of the river in Sudan, South Sudan and Ethiopia increases from June to September and decreases after that up to January. These sub-basins experience very low water levels from February to May. The river’s sub-basin in Egypt have less water compared to the rest of the country-basin units of the basin. The available water in the river’s country-basin unit in Eretria is also less than the rest because the sub-basin is not drained by one of the main tributaries of the Nile. The parts of the basin in Congo, Uganda, Kenya, Ruanda and Tanzania experience uniform and abundant water availability most of the year because these country-basin units are located in the equatorial region of the continent. Lake Chad’s country-basin unit in Niger and Algeria as well as Niger River’s country-basin unit in Algeria shows low water availability for six to seven months of the year. In the sub-basin of the river in Nigeria there is more water available than the country-basin units of the river in the other riparian countries for eight months of the year. Volta river basin is also one of the important river basin in West Africa. We observed variations in terms of water availability among the sub-basins of the river. There is more water available in the country-basin unit in Ghana than in the sub-basins of the river in Burkina Faso and Ivory Coast for large duration within the year. Generally, in most of the transboundary rivers’ country-basin units below the Sahara Desert and above the equatorial region more water is available from June to September than the rest of the year. In southern Africa except the Okavango sub-basin in Botswana and Limpopo river’s country-basin units in Zimbabwe & Botswana, which have more water compared to the rest of country-basin units in the region for few months, the other country-basin units of Okavango, Limpopo and Orange basins experience low but uniform available water for long duration of the year.

In Asia from July to September the available water in Ganges-Brahmaputra-Meghna and Mekong rivers’ country-basin units except in the sub-basin of the Mekong in China the available water is more than the rest of the months because during this period of the year natural runoff is relatively high. In Ganges-Brahmaputra-Meghna’s sub-basin in China the available water is lower than the rest of the country-basin units for most of the twelve-month period. The Indus River’s country-basin units in China, India and Pakistan shows relatively little variations among them in terms of water availability throughout the year. For few months of the year Aral Sea’s sub-basins in Kazakhstan and Uzbekistan have more available water than the rest of the country-basin units. Other country-basin units of transboundary water resources like the Aral Sea’s sub-basins in Kyrgyzstan, Afghanistan & Tajikistan, Hari/Harirud River’s sub-basin in Afghanistan & Iran, Obxx & Shu sub-basins in Kazakhstan, sub-basins of Traim River, Ili/Kunes He in China and Helmand River’s country-basin units in Afghanistan have relatively less variation in terms of available water throughout the year. Amur River which is shared between China and Russia have less available water from January to march while during the rest of the year water is abundant. The available water in the sub-basin of the river in Russia is more than the available water in the river’s country-basin unit in China. Donx, Volga, Jenisej/Yenisey & Obxx sub-basins in Russia have more available water most of the year than their sub-basins in China, Kazakhstan and Mongolia.

The country-basin units of the Tigris-Euphrates/Shatt al Arab in the Middle East shows little variations in terms of water availability throughout the year. From January to April more available water is observed in the country-basin units of the river in Turkey, Syria, and Iraq. This is not the case in the river’s sub-basin in Iran. This availability extends to June for the sub-basin in Iraq while reduces after April in the river’s country-basin units in Syria and Turkey.

Most of Western Europe’s transboundary river basins have less water compared to those eastern part of the continent for most months of the year. Majority of these transboundary river basins in Eastern and Western Europe experience less


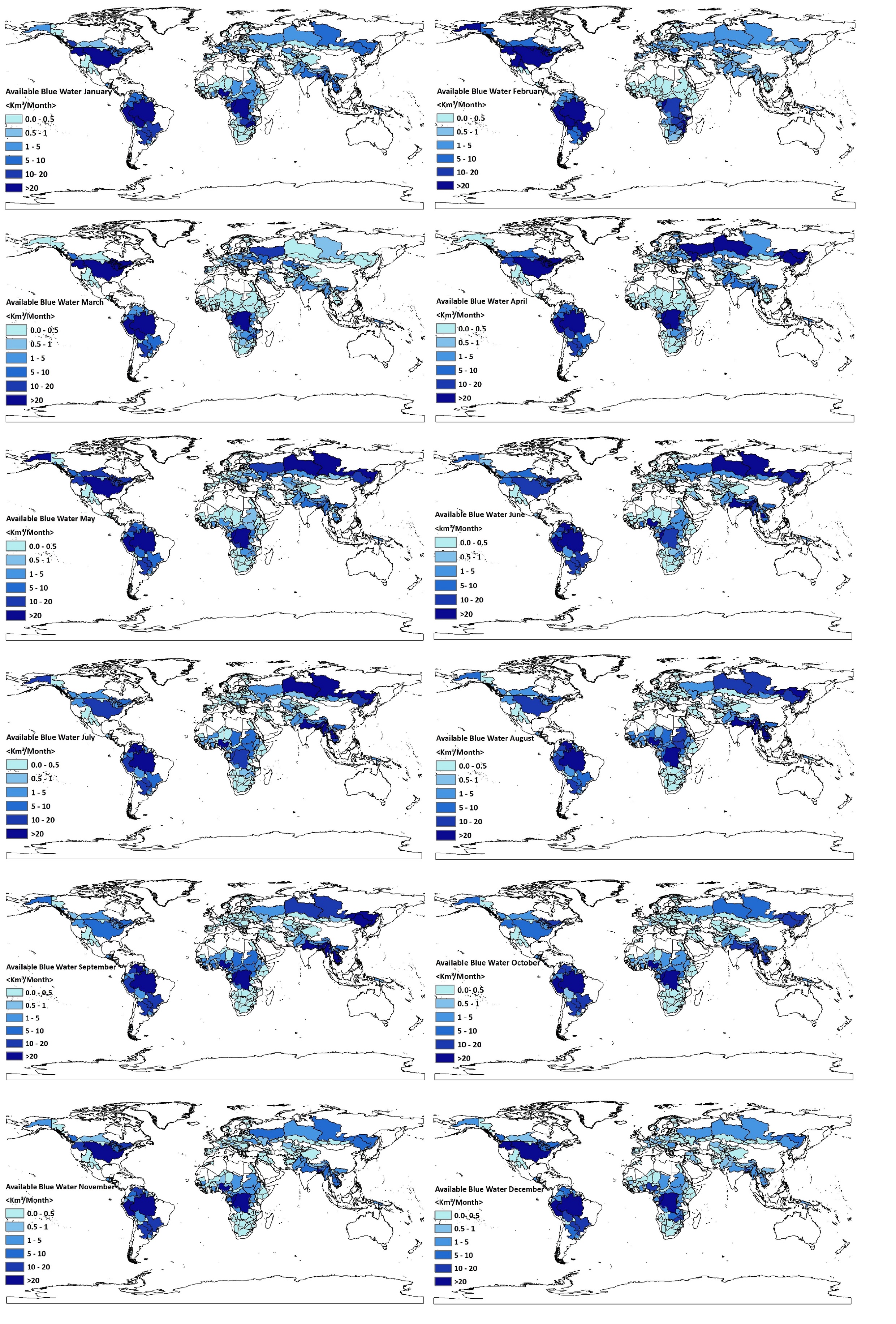


**Figure S2.** Monthly available blue water per country-basin unit of transboundary basins in km^3^/month. Period: 1996–2005. The available blue water within each country-basin unit was determined by adding the accumulated actual runoff and water footprint within each country-basin unit. These maps were generated with ArcGIS 10.2 for desktop (<http://www.esri.com/sofware/arcgis>).

water availability from July to September compared to the other months of the year. Generally, the differences in terms of available water among the country-basin units in these river basins are not very much.

In North America, the available water in Yukon River’s sub-basin in Canada, Colorado and Rio Grande country-basin units in United States and Mexico is lower from July to August than the rest of the months. Their discharge is also less than most of country-basin units of the rest of the transboundary river basins in the continent. Country-basin units of the Mississippi, Nelson-Saskatchewan, St. Lawrence, Columbia, Yukon sub-basin in United States have relatively high available water throughout the year.

In South America the sub-basin of the Amazon in Brazil has high and fairly constant available water throughout the year. The river’s country-basin units in Colombia, Peru, Bolivia and Venezuela have relatively less available water and show more disparity in terms of water quantity from month to month than the river’s sub-basin in Brazil. The La Plata river basin crossing Brazil, Paraguay, Uruguay, Bolivia and Argentina have also abundant water taking in to account upstream blue water consumption.

In a nutshell, the variations among the country-basin units of the transboundary river basins in terms of available water might be due to high upstream population count and/or density, high upstream water footprint and tributary rivers’ discharge difference amongst the sub-basins.

**Monthly Blue Water Scarcity by Country-Basin Spatial Unit**

Water scarcity in this article is defined as the ratio of water footprint to water availability^5,28^.Sub-basins’ water scarcity values obtained are different depending on the spatial & temporal resolution as well as on how water footprint & water availability are defined. In addition, they also influenced by the amount of sub-basins’ water assigned for maintaining ecological integrity. In the following paragraphs the water scarcity in the country-basin units of the main transboundary river and lake basins of Africa, Europe, South America and North America are described at monthly time step. The maps showing the water scarcity per country-basin unit are shown in **Fig. S3** and the water scarcity values of the sub-basins can be found in **Table S4.**

In Africa the country-basin units of the Nile river basin in Ethiopia, Sudan, South Sudan, Eretria and Egypt water is extremely scarce from February to March. This scarcity continues to April for the country-basin unit of the river basin in Sudan. While for the river’s sub-basin in Egypt this scenario continues throughout the year. This might be because Egypt is overly reliant on the Nile for industrial, domestic and agricultural water consumption. The sub-basin in Eritrea also goes through severe water scarcity most of year which might be due to the fact that it is one of the sub-basin in arid upstream riparian country drained by tributary of the Nile with low discharge. The sub-basin of the Nile in Ethiopia experience severe water scarcity in February and March while for the rest of the year the sub-basin experience low water sacarcity. The river’s sub-basins in the equatorial region spends most of the year under low water scarcity. The Juba-Shibeli River’s sub-basin in Somalia suffers from severe water scarcity for seven months of the year and low for the rest while the sub-basin of the river in Ethiopia is under low water scarcity for ten months and severe water scarcity for the remaining months of the year. The country-basin units of transboundary river and lake basins in Africa located south of the Saharan desert and above the equatorial region such as Lake Chad, Niger, Senegal, Cross, Oueme, Sanaga, Sassandra, Cavally, St. John (Africa), Gambia, Corubal, Little Scarcies, Mana-Morro, St. Paul, Cestos, Corubal, Geba and Moa rivers endures severe water scarcity from February to May. In Senegal River’s sub-basin in Mauritania severe scarcity continues up to the month of July. For Lake Chad’s and Niger River’s sub-basins in Algeria severe water scarcity prevails throughout the year. In Southern Africa Orange river’s country-basin units in Namibia, Lesotho, South Africa, and Botswana experience severe water scarcity for most months of the year, moderate to significant water scarcity for some of the months of the year and low blue water scarcity for at least one month of the year. The other main border crossing river in southern Africa is the Limpopo River. Its sub-basins face low water scarcity from January to June and significant to severe water scarcity from June to December. On the other hand, the country-basin units of Congo, Zambezi and Nile sub-basin in equatorial Africa as well as the country-basin units of the Okavango in Namibia and Botswana experience low blue water scarcity throughout the year.

In Asia Indus River’s country-basin units in India and Pakistan, the sub-basins of Ganges-Brahmaputra-Meghna in Bangladesh and India experience extreme water scarcity from January to April. In the following months from May to December the Ganges-Brahmaputra-Meghna’s country-basin units experience low to moderate water scarcity while the Indus River’s sub-basins face water scarcity all-round the year. The Indus River’s sub-basin in India experience severe water scarcity for seven months and moderate water scarcity for the rest. Its sub-basin in Pakistan experience one month moderate, eight severe and for the remaining significant water scarcity. The Indus in China, Ganges-Brahmaputra-Meghna in China and Nepal are the exceptions which experience low and moderate water scarcity for long duration of the year. In the country-basin units of the Mekong River in Laos and China water scarcity is very low for most of the year but parts of the basin in Cambodia and Thailand [encounter](http://www.freethesaurus.com/encounter) water scarcity from February to April. Jenisej/Yenisey and Hrun rivers’

**
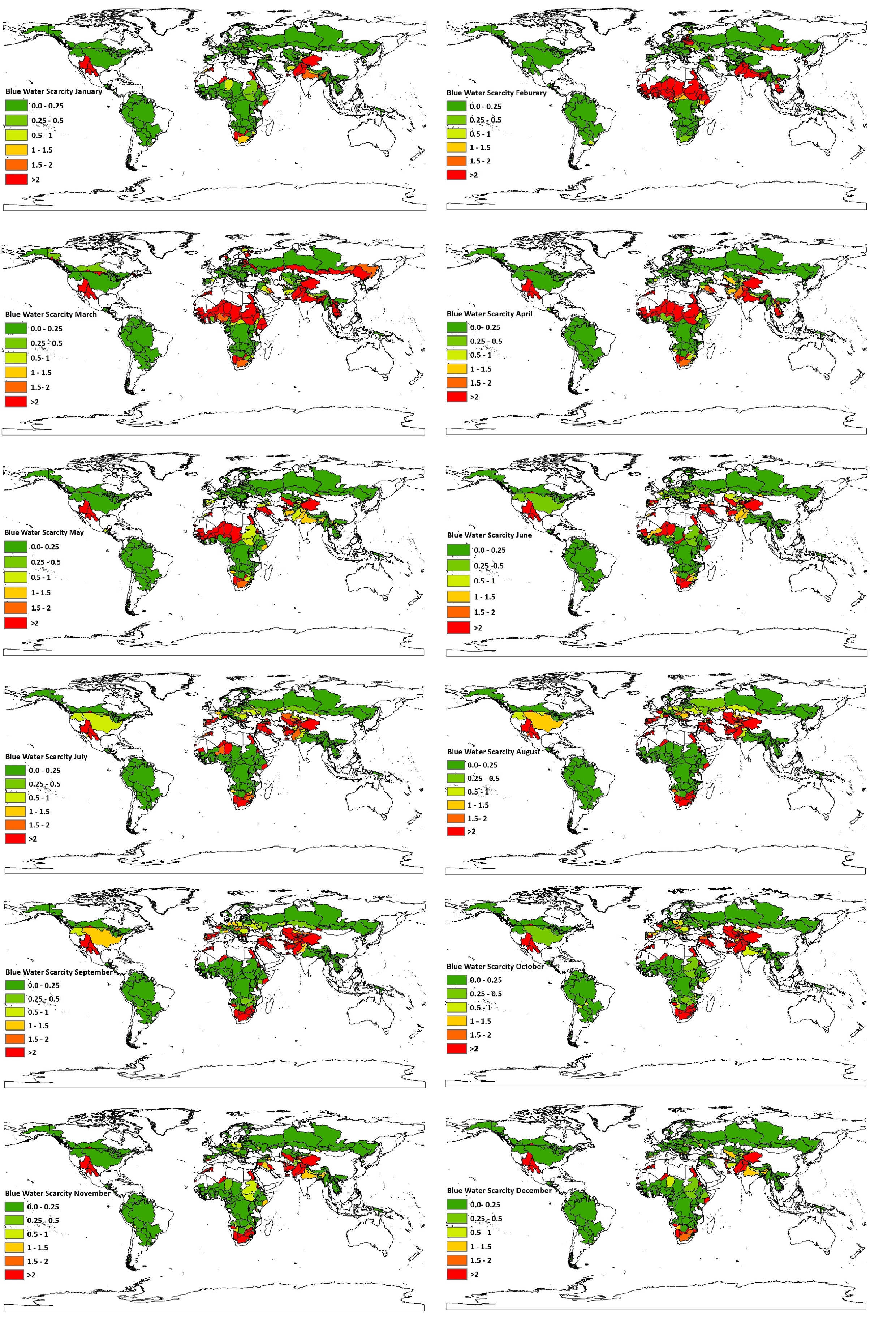
Figure S3.** Monthly Blue water scarcity per country-basin unit of transboundary basins. Period: 1996–2005. Blue water scarcity at country-basin mesh spatial resolution is defined as the ratio of the blue water footprint to the available blue water within each sub-basin. Maps generated with ArcGIS 10.2 for desktop (<http://www.esri.com/sofware/arcgis>).

country-basin units in Mongolia live through water scarcity at least one month of the year while these rivers’ country-basin units in Russia are under low water scarcity throughout the year. Obxx River’s sub-basin in Kazakhstan also [go through](http://www.freethesaurus.com/go+through) water scarcity one month of the year. In the sub-basins of the Amur River shared by Russia, Mongolia and China significant water scarcity prevails on the side of the river in Russia and severe in the part of the river basin located in China and Mongolia for at least for one month of the year. The sub-basin in Mongolia is under moderate water scarcity for an additional one month.

Pu Lun T'o river’s sub-basins in China and Mongolia experience significant to severe water scarcity for nine months. Jenisej/Yenisey, Obxx and Volga country-basin units in Russia face no water scarcity throughout the year. Tarim River in China is under severe water scarcity for most of the year. The Aral Sea in Uzbekistan and Kazakhstan suffers from severe water scarcity for seven and four months respectively. On the contrary its sub-basin in Afghanistan endures low water scarcity for eight months.

In the Middle East Tigris-Euphrates/Shatt al Arab river basin in Iran go through significant to severe water scarcity for nine months of the year. The river’s sub-basin in Iraq and Syria feels the impact of significant to severe water scarcity for seven months of the year. The part of the river basin in Turkey undergoes significant to severe water scarcity for five months of the year. This river’s three country-basin units experience water scarcity at least for six months.

In Eastern Europe Vuoksa River’s country-basin unit in Finland, Dnieper’s sub-basins in Belarus and Russia suffers from severe water scarcity at least one month of the year. Danube River’s sub-basin in Hungary faces significant water scarcity for three months of the year whereas the sub-basin in Romania is under moderate water scarcity for a month.

In Western Europe Seine River’s country-basin unit in France experience severe water scarcity from August to October and moderate scarcity during the month of July. The country-basin units of Guadiana, Duro, Tagu, Ebro, Lima river basins shared by Portugal and Spain suffers from severe water scarcity for four to six months within a year. The rest of the country-basin units of transboundary river basins in Western Europe experience low water scarcity for most of the year.

| **Month** | **Number of country-basin units facing low, moderate, significant and severe water scarcity during each month of the year** | | | | **Number of people (in millions) facing low, moderate, significant and severe water scarcity during each month of the year** | | | |
| --- | --- | --- | --- | --- | --- | --- | --- | --- |
|  | **Low water**  **scarcity** | **Moderate**  **water scarcity** | **Significant**  **water scarcity** | **Severe water**  **scarcity** | **Low**  **water**  **scarcity** | **Moderate**  **water scarcity** | **Significant**  **water scarcity** | **Severe water**  **scarcity** |
| **January** | 501 | 9 | 3 | 47 | 1,774.27 | 16.05 | 427.24 | 300.14 |
| **February** | 426 | 4 | 3 | 127 | 1,295.63 | 6.66 | 0.03 | 1,215.38 |
| **March** | 323 | 5 | 4 | 228 | 1,207.99 | 32.0 | 72.79 | 1,204.96 |
| **April** | 396 | 6 | 8 | 150 | 1,499.93 | 33.53 | 174.0 | 811 |
| **may** | 439 | 11 | 2 | 108 | 1,717.20 | 543.99 | 10.01 | 246.50 |
| **June** | 446 | 23 | 5 | 86 | 2,029.71 | 258.22 | 8.89 | 220.88 |
| **July** | 437 | 13 | 23 | 87 | 2,070.50 | 28.83 | 162.78 | 255.59 |
| **August** | 424 | 14 | 13 | 109 | 1,947.43 | 117.04 | 30.58 | 422.65 |
| **September** | 423 | 13 | 16 | 108 | 1,853.03 | 227.00 | 30.60 | 407.07 |
| **October** | 443 | 10 | 11 | 96 | 1,984.72 | 30.20 | 36.29 | 466.49 |
| **November** | 467 | 13 | 8 | 72 | 1,660.22 | 466.00 | 8.12 | 383.36 |
| **December** | 479 | 12 | 9 | 60 | 1,715.73 | 464.22 | 28.85 | 308.90 |

**Table S1.** Number of country-basin units and Number of people (in millions) facing low, moderate, significant and severe water scarcity during each month of the year. Period: 1996–2005

In North America the Mississippi river’s country-basin units undergo moderate water scarcity for three months from July to September. The part of the drainage in Canada experience severe water scarcity for ten months of the year. The Colorado, Rio Grande, Yaqui, country-basin units in the United States and Mexico suffers from extreme water scarcity all year round. Nelson-Saskatchewan and St. Lawrence country-basin units in United States and Canada are under low water scarcity throughout the year.

In South America all the transboundary river basins’ country-basin units experience low water scarcity throughout the year. This might be due to the fact that in the continent’s transboundary river’s country-basin units available water is abundant compared to water footprint.

Quarterly averaged water scarcity per country-basin units was determined to observe the seasonal variation of water scarcity among each transboundary river and Lake Basin’s country-basin units. This is important because the available water and footprint might show significant differences from season to season. The maps showing seasonal water scarcity per country-basin unit are shown in **Fig.2** and the water scarcity values of the sub-basins can be found in **Table S5.**

From January to March the main border crossing river and lake basins below the Sahara Desert above the equatorial region in Africa such as large part of the Nile, Juba-Shibeli, Volta, Lake Chad, Niger, Senegal, Cross, Oueme, Sanaga, Sassandra, Cavally, St. John (Africa), Gambia, Corubal, Little Scarcies, Mana-Morro, St. Paul, Cestos, Corubal, Geba and Moa rivers experience severe water scarcity throughout their country-basin units. While the sub-basins of Congo, Ogooue, Zambezi and the country-basin units of the Nile River in the equatorial countries are under very low water scarcity. In southern Africa Limpopo River’s sub-basin in Botswana endures extreme water scarcity during this season. On the contrary the river’s country-basin units in the other riparian countries are under low water scarcity. Sub-basins of Orange River basin in South Africa faces moderate water scarcity while its country-basin units in Namibia and Botswana undergo severe water scarcity at this time of the year. In the remaining country-basin units water needs are fulfilled while keeping the water reserved to sustain the ecological integrity of the sub-basins untouched. In Asia during this season the sub-basins of the Indus in India & Pakistan, Ganges-Brahmaputra-Meghan’s sub-basins in Bangladesh & India, sub-basins of the Mekong in Cambodia & Thailand, Traim river basin in China, Red/Song Hong river in China & Vietnam as well as Jenisej/Yenisey and Har Us Nur sub-basins in Mongolia are the main country-basin units that go through severe water scarcity. The sub-basin of the Amur River in Mongolia shows moderate water scarcity while the part of the river in China faces significant water scarcity. Other country-basin units like Pu Lun T'o in China and Oral/Ural river basin in Kazakhstan & Russia also live this season under significant water scarcity. Water scarcity level within Amur, Volga, Obxx and Jenisej/Yenisey rivers’ country-basin units in Russia is very low. Indus and Ganges-Brahmaputra-Meghan Rivers’ sub-basins China are also among the country-basin units which do not experience scarcity levels that threatens human and environmental water requirements. During this period of the year the country-basin units of Dnieper, Daugava, Lielupen, Gauja, Neman and Parnu experience significant to severe water scarcity in Eastern Europe. Country-Basin units of transboundary river basins in Western Europe experience low water scarcity for the duration of this season. In North America the Colorado, Yaqui & Rio Grande country-basin units undergoes significant water scarcity in United States and severe water scarcity in Mexico. Nelson-Saskatchewan River’s sub-basin in United States also found to be under significant water scarcity during this season. The transboundary basins of South America are unaffected by water scarcity during this period due to the fact that these rivers’ discharges are high compared to water footprints within the basins.

From April to June in the Nile River’s sub-basin in Ethiopia there is enough water to satisfy human and environmental needs while in the part of the river basin in Sudan, South Sudan and Eritrea the water scarcity level eases from severe to significant. On the other hand, the river basin’s country-basin unit in Egypt remains under severe water scarcity. Except for Niger’s sub-basin in Cameron & Nigeria, Volta & Komoe in Ivory Coast, Juba-Shibeli in Ethiopia the other transboundary river and lake basins’ country-basin units below the Sahara Desert and above equatorial Africa remain experiencing severe water scarcity. In southern Africa the water scarcity level of the Orange River’s sub-basin in South Africa increased from significant to severe. The sub-basin of Orange River in Namibia and Limpopo sub-basin in Botswana continues to be under severe water scarcity during this season. In the country-basin units of Okavango, Congo, Sabi, Pungwe, Kunene, Etos, and Zambezi as well as in the sub-basins of the Nile in Congo, Ruanda, Kenya, Tanzania and Uganda the low water scarcity status from January to March continues during this period too. In Asia during this season in the Indus river’s sub-basin in India the water scarcity level decreases from severe to significant while in the sub-basin of the river in Pakistan the scarcity level reduces to moderate water scarcity. The Helmand River’s sub-basin in Afghanistan and Aral Sea’s sub-basin in Uzbekistan go through significant and severe water scarcity respectively during this season. In the Mekong river basin water scarcity level in the river’s sub-basins Cambodia and Thailand reduced from severe to significant. The country-basin units of Jenisej/Yenisey, Amur, Pu Lun T'o and Obxx in Mongolia, China and Russia experience low water scarcity during this time of the year.

In eastern and western Europe all the sub-basin of the transboundary river basins within each riparian’s sovereign boarder experience low water scarcity. In North America the water scarcity in the sub-basins of Colorado, Yaqui and Rio Grande intensifies from significant to severe while these rivers country-basin units in Mexico remains under severe water scarcity during this time of the year too. The other country-basin units of North America’s transboundary basins remain to be under low water scarcity. In South America all the transboundary basins spend this season under low water scarcity throughout their country-basin units as well, mainly due to abundance of available water compared to water footprint.

From July to September except Senegal River’s sub-basin in Mauritania, sub-basin of Lake Chad & Niger river in Algeria, Juba-Shibeli in Somalia and the Nile’s sub-basins in Egypt & Eritrea all the other country-basin units of transboundary rivers below the Sahara Desert and above the equatorial region experience low water scarcity. In the equatorial and southern Africa, the Congo River’s country-basin units, sub-basin of the Nile in the Tanzania, Kenya, Rwanda, Congo & Uganda as well as the sub-basins of the Zambezi & Okavango are under low water scarcity during this time of the year. On the other hand, the country-basin units of the Orange, Limpopo, and Sabi in the southern Africa suffers from severe water scarcity. In Asia Ganges-Brahmaputra-Meghna in India and Bangladesh have enough water during this season to satisfy both environmental and human water needs. The Indus river basin in India during these three months experience low water scarcity on the contrary the river’s sub-basin in Pakistan endures severe water scarcity. Helmand River’s sub-basin in Afghanistan & Iran, Aral Sea’s sub-basins Uzbekistan, Kazakhstan & Kyrgyzstan and Ili/Kunes He & Pu Lun T'o rivers’ sub-basins in China are also under severe water scarcity. Throughout this period all country-basin units of the Mekong River have enough water available for consumption without compromising environmental water requirement. In the Middle East the country-basin units of the most important transboundary river basin, Tigris-Euphrates/Shatt al Arab are severely water scarce. In Western Europe Duro, Tagu, Guadiana, Lima in Spain and Portugal as well as Seine in France bear severe water scarcity during this period of the year. Most border crossing river basins in Russia and Eastern Europe have enough water during this time of the year. In North America the sub-basin of the Mississippi river in Canada is under severe water scarcity while in the sub-basin of the river in the United States the environmental and human water needs are satisfied. The Colorado, Yaqui & Rio Grande in the United States and Mexico continues to be under severe water scarcity during this time of the year too. In South America, because water is abundant relative to water footprint, the available water is enough to satisfy human water needs and maintain the ecological integrity of all the country-basin units of the continent during this time of the year as well.

From October to December the sub-basins of Juba-Shibeli in Somalia and Niger river’s & Lake Chad’s sub-basins in Algeria as well as sub-basins of the Nile in Egypt are the main transboundary rivers’ and lakes’ sub-basins that goes through severe water scarcity above the equatorial region in Africa. In the equatorial region the continent’s transboundary river basins’ country-basin units are under low water scarcity. In Southern Africa the country-basin units of Orange, Limpopo, Sabi, and Cuvelai/Etosha’s sub-basin in Namibia spend this season under severe water scarcity. In the Middle East Kura-Araks in Azerbaijan and the sub-basins of Tigris-Euphrates/Shatt al Arab in Syria are under significant water scarcity. On the other hand, Tigris-Euphrates/Shatt al Arab sub-basin in Iran suffers from severe water scarcity. In the river’s sub-basin in Turkey there is enough water to satisfy human and environmental water requirements whereas the country-basin unit in Iraq experiences moderate water scarcity. The sub-basins of the Indus river basin in Pakistan & India, Helmand River’s sub-basin in Iran & Afghanistan, Tarim in China, sub-basins of Aral Sea in Uzbekistan & Kyrgyzstan, Hari/Harirud in Iran are the major sub-basins in Asia that endure severe water scarcity throughout this season. In all of the continent’s other river and lake basins’ country-basin units there is enough water available for human consumption as well as for ensuring environmental sustainability. In Eastern and Western Europe all the country-basin units of transboundary river basins are under low water scarcity except Tagus/Tejo and Guadiana country-basin units in Spain which are under severe water scarcity this time of the year. In north and south America all river country-basin units except the sub-basins of the Colorado, Yaqui and Rio Grande river basins in United States and Mexico have enough water for maintaining environmental integrity as well as for satisfying agricultural, industrial and domestic water needs.
